# Effect of Divergent Selection for Intramuscular Fat Content on Muscle Lipid Metabolism in Chickens

**DOI:** 10.3390/ani10010004

**Published:** 2019-12-18

**Authors:** Lu Liu, Huanxian Cui, Siyuan Xing, Guiping Zhao, Jie Wen

**Affiliations:** 1 Institute of Animal Sciences, Chinese Academy of Agricultural Sciences, Beijing 100193, China; liulu_0907@126.com (L.L.); cuihuanxian78@126.com (H.C.); tcsxingsy@126.com (S.X.); 2 State Key Laboratory of Animal Nutrition, Beijing 100193, China; 3 Animal Breeding and Genomics, Wageningen University & Research, Wageningen 6708WD, The Netherlands

**Keywords:** chicken, selection, intramuscular fat, signaling pathway, differential lipid metabolism

## Abstract

**Simple Summary:**

Intramuscular fat is an important factor affecting meat quality and consumer acceptance. Appropriate increases in the intramuscular fat content contribute to the improvement of meat quality, and genetic selection is an effective method to increase the intramuscular fat content in chickens. In this study, chicken lines divergently selected for their intramuscular fat content were used to investigate the mechanisms behind differential intramuscular fat deposition. These results found in this study may contribute to the improvement of meat quality in chickens.

**Abstract:**

Intramuscular fat (IMF)—an important factor affecting meat quality—can be appropriately increased by genetic selection. Chicken lines divergently selected for IMF content were used in this study to investigate the mechanisms behind differential IMF deposition. Sixty 15th generation chickens were genotyped using the IASCHICK 55K single nucleotide polymorphism (SNP) chip. After quality control, 59 chickens and 36,893 SNPs were available for subsequent analysis. Population structure assessment indicated that the lines were genetically differentiated. Based on the top 1% paired fixation index values, three pathways were significantly (*p* < 0.05) enriched, and nine genes were considered candidate genes for differential IMF deposition. Differences between the lines in the expressions of representative genes involved in the above pathways were detected in 16th generation chickens. This study suggests that genetic selection for increased IMF in the pectoralis major muscle may enhance fatty acid synthesis, transport, and esterification, and reduce triglyceride hydrolysis. The peroxisome proliferator-activated receptor (PPAR) signaling pathway, glycerolipid metabolism, and fatty acid degradation pathway may have contributed to the differences in IMF deposition between the lines. These results contribute to the understanding of the genetic mechanisms behind IMF deposition, and the improvement of chicken meat quality.

## 1. Introduction

Meat quality plays an essential role in consumer acceptance, and intramuscular fat (IMF), deposited within the muscle tissue, is a widely acknowledged indicator of meat quality [1]. Numerous studies have explored the molecular mechanisms behind differential IMF deposition using different chicken breeds with typically different IMF contents [2,3,4]; however, the different genetic backgrounds of the chickens used makes explaining the inconsistent results of these studies difficult. As they have the same genetic origin, IMF-divergent selection lines are considered a good model for studying the molecular mechanisms behind IMF deposition.

Based on its heritability and adequate variability, it is possible to increase the IMF content through genetic selection. IMF selection programs have been performed in many livestock, including pigs [5], cattle [6], chickens [7,8], and rabbits [9]. In chickens, some divergent selection lines for highly heritable traits have been successfully bred, such as for body weight [10], abdominal fat [11,12], and the ultimate pH of the pectoralis major muscle [13]. However, the low to moderate heritability (0.12–0.16) of the IMF content in native Chinese chickens [7] increased the difficulty of genetic selection, resulting in few IMF selection lines being developed thus far.

An IMF selection program using a yellow-feathered chicken breed (Jingxing 100) has been undertaken by our research group since 2000. Our previous research showed that selecting for an increased IMF content resulted in lower shear-force values and improved meat quality in chickens after five generations of selection [8], thereby demonstrating the correlated phenotypic responses to the selection for increased IMF. However, the allele frequency and the expression of genes related to the target trait may have also changed during this long-term selection. In this study, meat-type chickens from the 15th (G15) and 16th (G16) generations of chicken lines divergently selected for IMF content were used to explore the correlated genomic response to divergent selection for IMF and to identify key functional genes involved in differential IMF deposition in chickens.

## 2. Materials and Methods

### 2.1. Ethics Statement

This study was conducted in accordance with the Guidelines for Experimental Animals established by the Ministry of Science and Technology (Beijing, China). All experimental protocols were approved by the Science Research Department in charge of animal welfare of the Institute of Animal Sciences, Chinese Academy of Agricultural Sciences (Beijing, China) (No. IAS2019-21). All birds were raised under the same recommended environmental and nutritional conditions. Basal diets were formulated based on the National Research Council (1994) requirements and the Feeding Standards of Chickens established by the Ministry of Agriculture, Beijing, China (2004).

### 2.2. Experimental Populations

The broiler lines used in this study were derived from the flock at the Institute of Animal Sciences of the Chinese Academy of Agricultural Sciences, which has been divergently selected for IMF content since 2000. The broilers in the two lines originated from the same base population of Jingxing yellow-feathered chickens (Jingxing 100, bred by Institute of Animal Science, Chinese Academy of Agricultural Sciences). In each generation, at least 3 male and 3 female chickens within the same family were slaughtered, and IMF content was measured (described below). The selection was based on the IMF mean within a family. The F Line was selected to increase the IMF, and the C line was a randomly-bred control line, as described by Zhao et al. [8].

### 2.3. Sampling

In G15, 30 birds from the F line and 30 from the C line were randomly selected at 98 days of age. Blood was collected from the wing veins of the chickens and stored with an anticoagulant until genomic DNA (gDNA) extraction and genotyping.

In G16, 8 birds randomly selected per line from the F and C lines. The pectoralis muscle samples were weighed, snap-frozen in liquid nitrogen, and stored at −80 °C until RNA isolation. The remaining pectoralis muscle tissues were removed, weighed, and stored at −20 °C until the measurement of the IMF, triglyceride (TG), phospholipid (PL), and total cholesterol (TCHO) contents.

### 2.4. Genotyping and Quality Control

gDNA was extracted from the blood samples from the G15 birds using the phenol-chloroform method. In total, 60 chickens were genotyped using the IASCHICK 55K single nucleotide polymorphism (SNP) chip (Supplementary Tables S4 and S5). Genomic data were derived from Liu et al. [14]. Before statistical analysis, the SNPs were pre-processed using PLINK software (v1.9) [15]. SNPs were selected based on SNP call rate of more than 95% and minor allele frequency (MAF) of more than 1%. The remaining SNPs were assigned to 1–28 chromosomes. Moreover, individuals were excluded due to sample call rate of less than 95%. Quality-controlled SNPs were annotated using the reference genome (Gallus_gallus_5.0, GCA_000002315.4).

### 2.5. Population Structure

Multidimensional scaling (MDS) analysis was conducted using PLINK (v1.07) software [15]. Independent SNPs were identified on all the autosomes, and SNPs with pairwise genotype r^2^ values of 0.2 or higher were removed. MDS components were acquired based on the pairwise identity-by-state matrix, and the relative kinship matrix was constructed from these independent SNPs, as described in detail by Sun et al. [16]. Based on the independent SNPs, the phylogenetic tree was constructed using TASSEL (v5.0) [17] and displayed using the Interactive Tree Of Life (iTOL) (https://itol.embl.de) [18].

### 2.6. Genetic Differentiation and Function Annotation

The genetic differentiation estimate, known as the pairwise fixation index *F*_ST_ [19], was calculated using GENEPOP software (v4.2) [20] using the quality-controlled SNPs. SNPs with top 1% *F*_ST_ values were identified as trait-related SNPs. Genes harboring these SNPs (if SNPs were not on genes, the closest genes around the SNP were selected, including upstream and downstream) were selected for pathway and functional enrichment analyses using KOBAS 3.0 [21] (http://kobas.cbi.pku.edu.cn). The significance level for pathway enrichment was set as *p* < 0.05.

### 2.7. Phenotype Measurement

The content of IMF, TG, and TCHO was conducted as reported previously [22]. The IMF content of the pectoralis major muscle was determined by extraction with anhydrous ether in a Soxhlet apparatus [23], and expressed as a percentage of the dry weight of the muscle sample.

The TG and TCHO contents of the pectoralis major muscle samples were measured using TG and TCHO assay kits (Nanjing Jiancheng Bioengineering Institute, Nanjing, China), and the PL content was measured using a PL assay kit (Leadman Biochemical Technology Co., LTD, Beijing, China). Pectoralis major muscle samples (about 2 g) from each chicken were homogenized with absolute ethanol at room temperature and centrifuged (1000 × *g*, 20 min). After centrifugation, the supernatant was used for TG, PL, and TCHO measurements. The absorbance for TG and TCHO was measured using a microplate reader at 510 nm, and the absorbance for PL was measured at 600 nm. The assays were performed according to the manufacturers’ instructions.

### 2.8. RNA Extraction

*Pectoralis major* muscle samples from all the selected G16 individuals were used for RNA extraction and subsequent quantitative real-time polymerase chain reactions (qRT-PCR). Total RNA was isolated from the pectoralis major muscle sample from each chicken using a total RNA kit (Tiangen, Beijing, China), according to the manufacturer’s instructions. After concentration, the purity and integrity were determined, and RNA samples with A260/A280 ratios between 1.8 and 2.0 were used for qRT-PCR.

### 2.9. qRT-PCR

The primers were designed using Primer Premier 6.0 software, and specificity was determined using BLASTN (Supplementary Table S1). The qRT-PCR analysis was performed after a reverse transcription reaction. cDNA was prepared through the reverse transcription of 2.0 μg of the total RNA from each sample using the FastQuant RT Kit (Tiangen, Beijing, China), according to the manufacturer’s instructions. The qRT-PCR reaction was performed in a total volume of 20 μL, consisting of 10 μL of 2 × iQTM SYBR Fast qPCR Master Mix (KAPA, Wilmington, MA, USA), 0.5 μL (10 mmol/L) of each primer and 1 μL of cDNA. Samples were amplified using the QuantStudio 7 Flex system (Applied Biosystems, Shanghai, China) to conduct 40 cycles (95 °C for 3 min, 95 °C for 3 s and 60 °C for 34 s). The amplification procedure was performed with 3 replicates for each sample, and a no-template negative control was also run in parallel. The collected data were analyzed using the 2^–*ΔΔCt*^ method [24], and all the results were normalized to the *18S* rRNA gene.

### 2.10. Statistical Analyses

SPSS version 22.0 (IBM Corp, Armonk, NY, USA) was used to test the significance of the differences between the groups using Student’s *t*-test. Confidence limits were set at 95%, and *p* < 0.05 (*) or *p* < 0.01 (**) was considered significant. Data are presented as the mean ± the standard error (SEM).

## 3. Results

### 3.1. Genotyping Statistics

A total of 60 chickens (30 chickens from each line) and 52,184 SNPs were genotyped using the IASCHICK 55K SNP chip. After quality control steps, the final data included 59 individuals and 36,893 SNPs for subsequent analysis.

### 3.2. Population Structure Analysis

**Figure 1:**
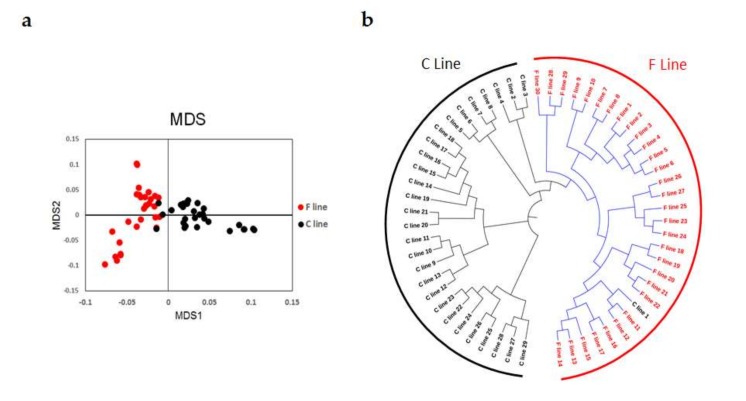
Population structure analysis of the 15th generation (G15) of chickens divergently selected for intramuscular fat (IMF) content, based on 55K single nucleotide polymorphism (SNP) chip data. (**a**) Multidimensional scaling analysis (MDS). (**b**) Phylogenetic tree. F line: selected for increased IMF; C line: randomly-bred control line.

Population structures were analyzed using MDS and phylogenetic tree analysis. MDS analysis was performed using the genotyped data, and the result was plotted using the first two principal components. This plot showed that the individuals from the two groups were clearly clustered into their respective lines (Figure 1a) and that individuals within each line had a closer genetic relationship than between the lines. The result of the phylogenetic tree analysis was consistent with the result of the MDS analysis (Figure 1b).

### 3.3. Genetic Differentiation and Function Annotation

**Figure 2:**
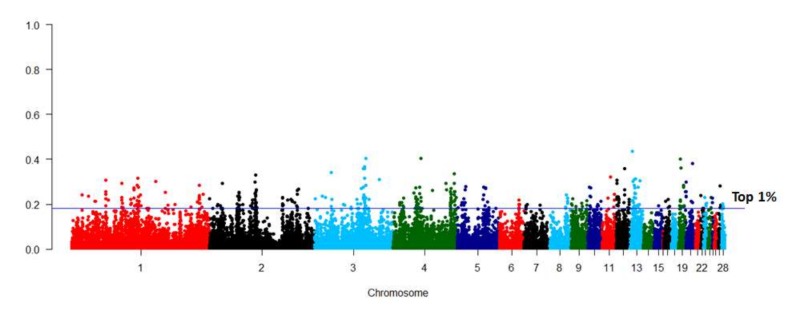
Manhattan plot of fixation index (*F*_ST_) value between the 15th generation (G15) of two lines of chickens divergently selected for intramuscular fat content. The blue line indicates the top 1% *F*_ST_ values.

The *F*_ST_ between the two lines was estimated using the 36,893 SNPs, which remained after quality control (Figure 2). The most significant SNPs were enriched on GGA1, GGA2, GGA3, and GGA4. There were 369 SNPs within the top 1% *F*_ST_ values, and these were identified as trait-related SNPs. Genes harboring these SNPs were selected for pathway and function enrichment analyses (Supplementary Table S2). Pathways associated with lipid metabolism were significantly enriched (*p* < 0.05), including the peroxisome proliferator-activated receptor (PPAR) signaling pathway, glycerolipid metabolism, and fatty acid degradation pathway (Supplementary Table S3). Nine genes, including acyl-CoA synthetase long chain family member 1(*ACSL1*), *PPARα*, acyl-CoA dehydrogenase long chain (*ACADL*), fatty acid binding protein 6 (*FABP6*), *FABP7*, phospholipid phosphatase 3 (*PLPP3*), pancreatic lipase related protein 1 (*PNLIPRP1*), membrane bound O-acyltransferase domain containing 1(*MBOAT1*), and aldehyde dehydrogenase 3 family member A2 (*ALDH3A2*), were enriched in these pathways, and were, therefore, considered important candidate genes for differential IMF deposition in chickens, based on their basic functions.

### 3.4. Phenotypic Differences in Pectoralis Muscle

**Figure 3:**
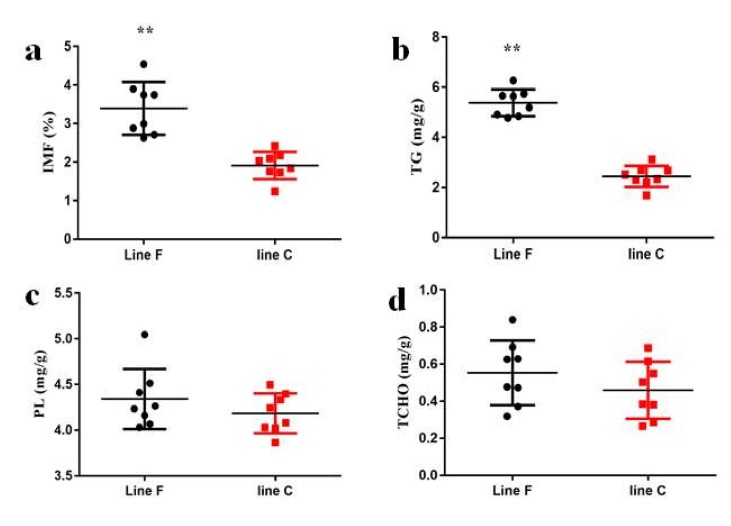
The (**a**) intramuscular fat (IMF), (**b**) triglyceride (TG), (**c**) phospholipid (PL), and (**d**) total cholesterol (TCHO) contents of the pectoralis major muscle tissue of 98-day-old 16th generation (G16) chickens from two lines divergently selected for IMF content. F line: selected for increased IMF (*n* = 8); C line: randomly bred control line (*n* = 8). ** *p* < 0.01.

As shown in Figure 3a, selection resulted in significantly higher IMF contents in the pectoralis major muscle tissues of the F line than that of the C line at 98 days of age (*p* < 0.01). Given that the TG, PL, and TCHO are the main components of the IMF [1], the differences in these components between the two lines were explored in this study. As shown in Figure 3b–d, the TG content was significantly higher in the F line than in the C line (*p* < 0.01), but there were no differences in either the PL or TCHO contents between the two lines (*p* > 0.05).

### 3.5. The Expression of Representative Genes Involved in Lipid Metabolism in Pectoralis Muscle Tissue

**Figure 4:**
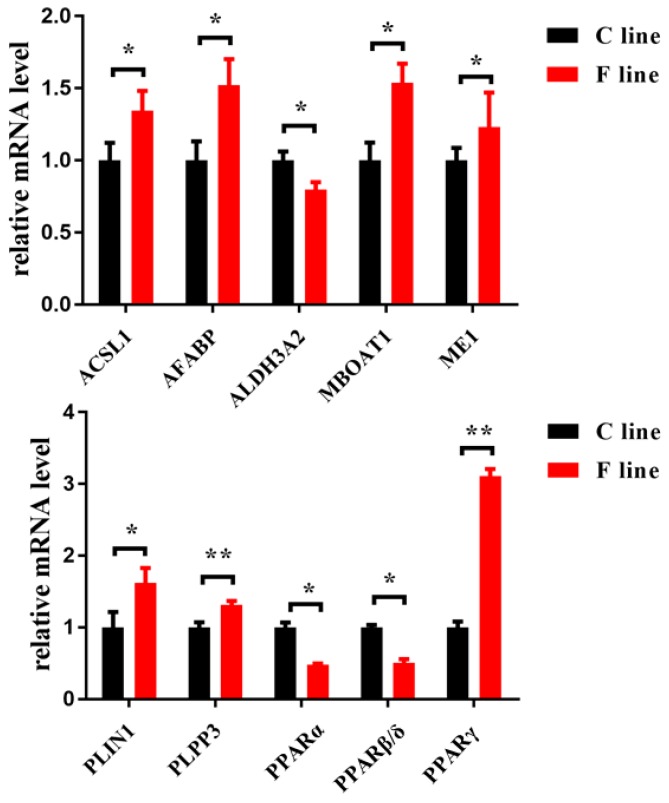
The expressions of representative genes involved in lipid metabolism in the pectoralis major muscle tissue of 98-day-old 16th generation (G16) chickens from two lines divergently selected for IMF content (* *p* < 0.05 or ** *p* < 0.01). F line: selected for increased IMF; C line: randomly bred control. (Abbreviations: *ACSL1*, acyl-CoA synthetase long chain family member 1; *AFABP*, fatty acid binding protein 4; *ALDH3A2*, aldehyde dehydrogenase 3 family member A2; *MBOAT1*, membrane bound O-acyltransferase domain containing 1; *ME1*, malic enzyme 1; *PLIN1*, perilipin 1; *PLPP3*, phospholipid phosphatase 3; *PPAR*, proliferator-activated receptor.).

To validate the candidate genes screened in the G15 chickens using the 55K SNP chips, the expression of representative genes in the two lines in G16 was determined using qRT-PCR (Figure 4). The expressions of *PPARα* and *ALDH3A2* in the pectoralis major muscle tissue were significantly lower in the F line than in the C line (*p* < 0.05), whereas the expressions of *ACSL1*, *MBOAT1*, and *PLPP3* were significantly higher than in the C line (*p* < 0.05 or *p* < 0.01). In addition, the expressions of several key genes involved in the PPAR signaling pathway were also explored, including *PPARβ/δ*, fatty acid binding protein 4 (*AFABP*), malic enzyme 1 (*ME1*), perilipin 1 (*PLIN1*), and *PPARγ*. Relative to the C line, the F line chickens had lower expressions of *PPARβ/δ*, but higher expressions of *AFABP*, *ME1*, *PLIN1*, and *PPARγ* (*p* < 0.05 or *p* < 0.01).

## 4. Discussion

Selection is an effective way to improve important economic traits in broilers, such as the growth rate and feed conversion ratio. Based on the heritability, chicken lines with divergent IMF contents could be achieved using conventional family-based selection. Indeed, the divergently selected chicken lines used in this study differed significantly in the IMF contents of their breast muscle tissue [7,8]. Given the limited accuracy of genetic evaluation using a phenotypic selection program, it was necessary to confirm the genomic response after multiple generations to this divergent selection for IMF.

### 4.1. Identified Genes and Pathways Related to Lipid Metabolism between Lines by Selection Signature Analysis

Genotyping data for the G15 chickens were obtained using 55K SNP chips covering 28 autosomal chromosomes. Population structure was assessed using MDS and phylogenetic tree analysis, which confirmed the effectiveness of the selection program and the different selection directions of the two lines. After long-term selection for the IMF content, the chromosome regions that harbor important genes for IMF deposition may exhibit different allele or haplotype frequencies [25]. The *F*_ST_ is a commonly used method to detect selection signatures between lines or breeds in domestic animals [26,27] and could be used as an additional source of information in genomic selection analysis [28]. According to the *F*_ST_ values determined in this study, there was moderate genetic differentiation between the lines. Based on the genes found to harbor trait-related SNPs (which had the top 1% *F*_ST_ values), the classic PPAR signaling pathway, glycerolipid metabolism, and the fatty acid degradation pathway were significantly enriched (*p* < 0.05), indicating that there were differences in the muscle lipid metabolism process between two the lines. Nine genes (*ACSL1*, *PPARα*, *ACADL*, *FABP6*, *FABP7*, *PLPP3*, *PNLIPRP1*, *MBOAT1*, and *ALDH3A2*) were found to have been enriched in the above pathways. These genes are involved in lipid metabolism and may be important candidate genes for differential IMF deposition. The above results indicated that long-term selection may affect the muscle lipid metabolism process in differentially selected lines.

### 4.2. PPAR Pathway Regulates Weakened Lipolysis and Enhanced Lipogenesis in F Line

To confirm this result, chickens in G16 were used to verify the differences in lipid deposition between the two lines. The phenotypic results indicated that the IMF content in the F line was significantly higher (*p* < 0.01) than in the C line at 98 days of age (breeding age), and that this may have been as a result of differential TG deposition. In addition, qRT-PCR was used to explore the expressions of the representative genes identified in the chickens using the 55k SNP chips. In the pectoralis major muscle tissue of the F line, *PPARα* was upregulated and *ACSL1* was downregulated, relative to the C line. PPARs are classical transcription factors involved in lipid metabolism and include three isotypes (PPARα, PPARβ/δ, PPARγ) with different tissue distributions and regulatory activities. *PPARα* is expressed mainly in tissues with a high capacity for fatty acid oxidation. In mammalian liver tissue, PPARα induces fatty acid oxidation through the activities of the mitochondria and peroxisomes, then increases acetyl-CoA production to provide energy [29]. Therefore, decreased *PPARα* expression in the F line suggests that lipolytic metabolism in the *pectoralis major* muscle tissue of these chickens was slower than in the C line chickens. *ACSL1* encodes a type of long-chain acyl-CoA synthetase that is involved in both lipid biosynthesis and fatty acid degradation. *ACSL1* is upregulated by PPARα ligands in the liver and is elevated by PPARγ agonists in the adipose tissue [30]. *ACSL1* overexpression can promote triglyceride accumulation in adipocytes [31]. A previous study showed that *ACSL1* enhanced the synthesis of triglycerides and resulted in higher IMF contents [32]. The results of this study were consistent with this interpretation, as the higher *ACSL1* expression in the F line than the C line coincided with the greater IMF deposition found in the former. These results indicated that weaker lipolysis and stronger lipogenesis processes may contribute to the higher IMF content found in the F line than the C line. To verify this hypothesis, the expressions of several genes associated with lipolysis and lipogenesis in the PPAR signaling pathway were explored. The expression of *PPARβ*/*δ* was downregulated, whereas *PPARγ* was upregulated in the F line. The effect of PPARβ/δ is similar to those of PPARα, which promotes energy dissipation, but opposite to those of PPARγ, which promotes energy storage [33]. PLIN1 was found to promote lipid droplet formation by activating the PPARγ signaling pathway [34]. Relative to the C line, the upregulated expression of *PPARγ* in the F line may directly increase *PLIN1* expression. Each PLIN family member likely performs a unique function in the regulation of lipid droplet dynamics [35], and PLIN1 plays a “switch” role in regulating lipolysis. Native PLIN1 may serve as a barrier, protecting droplet triglycerides from basal lipolysis, whereas phosphorylated PLIN1 could indirectly activate ATGL [36,37]. Previous studies have found that the expression of *PLIN1* was higher in muscles with high IMF contents [34,38]. In addition, *AFABP*, a negative regulator of mitochondrial fatty acid oxidation [39], was also upregulated in the F line. In avian, most of the NADPH used for fatty acid synthesis is generated by a malic enzyme (ME1) [40], and the expression of *ME1* is correlated with fat deposition in broiler chickens [41]. The upregulation of *ME1* in the F line indicated that fatty acid synthase was activated in these chickens. In the PPAR signaling pathway, lipolysis related genes were downregulated (*PPARα* and *PPARβ*/*δ*), and lipogenesis related genes were up regulated (*ACSL1*, *AFABP*, *ME1*, *PLIN1* and *PPARγ*) in the F line. These results demonstrated that the F-line chickens exhibited enhanced fatty acid synthesis, transport, and esterification processes, and reduced hydrolysis of triglycerides stored in lipid droplets, which may explain the higher IMF deposition in the F than C line.

### 4.3. Other Pathways Contribute to Increased IMF Deposition in F Line

In addition to the PPAR pathway, several other pathways were also involved in the differential IMF deposition between the two lines. *ALDH3A2* was enriched in the glycerolipid metabolism and fatty acid degradation pathway and is a classic target gene of PPARα, being involved in peroxisomal and mitochondrial fatty acid oxidation [42]. It has been reported that (−)-hydrocitric acid- reduced fat accumulation may be associated with an increase in the *ALDH3A2* expression level in chicken embryos [43]. The expression of *ALDH3A2* was significantly lower in the F line, indicating that the lipolysis process was reduced relative to the C line. The expression of another candidate gene, *PLPP3*, was also found to differ between the lines. The LPP3 substrate phosphatidic acid (PA), is an intermediate in the synthesis of triglycerides from dietary fats and carbohydrates [44]. LPP3, which is encoded by the *PLPP3* gene, is involved in the penultimate step of triglyceride synthesis and has the ability to dephosphorylate PA, thereby generating the triglyceride precursor diacylglycerol [45]. Upregulated *PLPP3* expression in the F line indicated that increased triglyceride synthesis from dietary fats and carbohydrates was taking place in these chickens. In addition, *MBOAT1*, which was enriched in glycerolipid metabolism pathway, also showed higher expression in the F line. MBOAT1 has been reported to be associated with phosphatidylcholine metabolism [46] and large intestine lipid metabolism [47]. An upregulation of *MBOAT1* may, thus, also be associated with differences in IMF deposition between the two lines.

### 4.4. Necessity for Further Studies

The possible mechanisms of differential IMF deposition by selection have been revealed in this study. However, there were still some limitations to this research worth discussing. There was no doubt that *F*_ST_ is an effective method to detect selection signatures. However, it is unavoidable to yield false signatures depending on a single approach. Na et al. [25] tried to use *F*_ST_ and hapFLK to explore selection signatures for chicken abdominal fat deposition, while there was no overlap between two methods. Therefore, the relative gene expression was used to verify the reliability of the results in this study. However, by the limitation of sample size and chip density, it is necessary to explore mechanisms of differential IMF deposition by enlarging sample size and sequencing depth in further studies.

## 5. Conclusions

Divergent selection for the IMF content led to differential IMF deposition in the pectoralis major muscle tissue between the two lines. Genetic selection for increased IMF in chickens’ pectoralis muscle tissue may be achieved through the upregulation of the expression of genes involved in fatty acid synthesis, transport, and esterification, as well as the downregulation of the expression of genes involved in lipolysis in the PPAR signaling pathway. In addition, several other pathways also contribute to this process, including glycerolipid metabolism and the fatty acid degradation pathway. The regulatory networks involved in these pathways and the expressions of these genes may be responsible for the increased IMF content in the F line chickens after multiple generations of selection.

## Supplementary Materials

The following are available online at https://www.mdpi.com/2076-2615/10/1/4/s1, Table S1: The specific primers for qRT-PCR in this study. Table S2: Candidate genes based on the top 1% *F*_ST_ value. Table S3: The enriched pathways based on candidate genes. Table S4: Sequencing sample number and grouping. Table S5: Genotype data by IASCHICK 55K SNP chip.
